# To queue or not to queue: Facility managers and mental health care users’ experiences of integrated health care in a rural South African district

**DOI:** 10.1177/00207640231177832

**Published:** 2023-05-28

**Authors:** Divan Rall, Leslie Swartz

**Affiliations:** Department of Psychology, Stellenbosch University, Matieland, Stellenbosch, South Africa

**Keywords:** Eastern Cape, public mental health care, rural mental health care, South Africa

## Abstract

**Background:**

A key feature of South Africa’s state health care strategy since 1994 has been the development and expansion of services towards integrated health care at primary health care level. Within the new system, emphasis has been on the integration of patients with mental health care needs with other patients where multiple health conditions and needs would be addressed simultaneously. As part of a larger study into mental health care in a predominantly rural district, we investigated the experiences of facility managers and mental health service users in rural clinics within the system of care. We were interested both in their views as to the advisability of the integrated model and the ways in which they managed any challenges they may have experienced within the system at local level.

**Methods:**

Data were collected through once-off semi-structured interviews with facility managers and mental health care service users to gather qualitative information. Narratives were transcribed and translated into English. Transcriptions were imported to Atlas.ti 22 and analysed through Thematic Analysis.

**Results:**

The integration of mental health care into routine primary health services poses challenges to treatment delivery and to patients who come for treatment. Our study also suggests resegregating mental health care as a possible solution to facilitate service delivery and treatment to service users.

**Conclusion:**

This research provided first insights into facility managers’ and service users’ views of integrated mental health care at primary health care level in this district. While mental health care services have been expanded and integrated into primary health care over recent years, the system may not have been as streamlined as in other parts of the country. The integration of mental health into primary health care can pose various challenges to facilities, health care providers, and mental health service users. Managers in these constrained circumstances have observed that resegregating mental health care from physical treatment, as in the past, may be deemed more effective for health care provision and reception. Generalised integration of mental health treatment with physical care should be approached with caution unless there is wider scale provisioning and greater organisational change.

## Background

As South Africa moved to a democracy in 1994, there were concomitant changes in all aspects of society, including health care. A key feature of state health care strategy since 1994 has been the development and expansion of services at primary health care level. In common with many countries with colonial histories, health expenditure in South Africa was skewed towards tertiary hospital services in major urban centres; the principle of equity in health care required an expansion of community-based health care provision across the entire country.

The concept of primary health care (PHC) has been defined as: *an essential health service that is the first contact care for individuals and communities that is universally accessible and can provide comprehensive treatment (i.e. providing promotive, preventive, curative, supportive and rehabilitative care) to the health care service users as close as possible to their everyday environment. PHC is an approach to health that aims at organising and adapting services to the needs of the population served in facilitating the highest possible level of health and well-being (Muldoon et al., 2006)*.

With South Africa’s divided and discriminatory history, this inclusive approach rapidly became intertwined with broader political imperatives concerning integration in society as a whole. Not only were services to be fully integrated in terms of race, but a vision for services was that these would be offered in as integrated a way as possible. The term ‘one-stop shop’ (Hlongwa & Sibiya, 2019) has been used to describe the ideal PHC system, with patients being able to access all their health needs (preventive, promotive, and curative and across all health conditions) ideally in one place and at one time.

Historically, mental health (MH) services at PHC level, where available, had been offered as a specialist service within PHC, commonly staffed by specially trained MH (psychiatric) nurses, supported by psychiatrists who would visit clinics intermittently. There were specific days on which MH services were offered in what has been termed a ‘silo’ approach to service delivery. Within the new integrated system, emphasis was on integration of patients with MH care needs with other patients into what was conceived of as a streamlined service where multiple health conditions and needs would be addressed simultaneously (Dookie & Singh, 2012; Phaswana-Mafuya et al., 2008). Ideally, this desegregation of those with obvious MH care needs from other patients would lead to de-stigmatisation, with MH care patients being seen similarly to other patients with acute and chronic health conditions, such as colds, diabetes and hypertension. Indeed, a proportion of those with hypertension and diabetes would have MH needs and concerns as well, and these, it was envisaged, would be dealt with integratively.

There is no question that in certain areas of South Africa impressive gains have been made integrating MH care into PHC (Lund et al., 2010; Petersen et al., 2009). However, nearly 30 years into democracy, the question arises as to how the ideologies of integration of MH and PHC are understood and managed by frontline health care workers and patients in rural parts of South Africa, which have not been studied in relation to this issue. The Eastern Cape province has been one of the poorest parts of the country for more than a decade (Statistics South Africa, 2017). As part of a larger study into MH care in an area of the province with a predominantly rural population, we report here on experiences with the system of care of facility managers and MH service users in rural clinics. We were interested both in their views as to the advisability of the integrated model and the ways in which, at local level, they managed any challenges they may have experienced with the system.

## Research methods

### Study setting

The study was conducted in the public health care sector in the Dr Beyers Naude Local Municipality (DBNLM) area in the Eastern Cape province. DBNLM had a population of 82,197 in 2016 (Statistics South Africa, 2018). The area is made up of towns, namely, Graaff-Reinet, Nieu Bethesda, Aberdeen, Rietbron, Willowmore, Jansenville, Steytlerville and Klipplaat, and a number of smaller settlements and surrounding farms. Data were collected from five PHC clinics, and one community day care facility in the DBNLM district. DBNLM has not been studied in terms of MH care access. The area is familiar to the first author, who works and resides in the region.

### Study design

As part of a larger investigation into MH issues in PHC in DBNLM, we used an exploratory qualitative design for this sub-study. Exploratory studies seek to investigate and obtain insight into a situation or social phenomena. Exploratory approaches are particularly useful when limited information exists on the subject being studied. Qualitative strategies aim to comprehensively describe participants’ experiences (Willig, 2013). This approach is especially well suited to studies where little is known about the context and the phenomenon being studied.

### Participants and data collection

The participants for this sub-study were facility heads/managers at their respective establishments and adult mental health care users (MHCUs) at these facilities (see Table 1 for study population description). Participants were recruited though advertisement of the project at the health care centres. Following ethical approval, participants who volunteered participation were telephonically contacted and appointments to meet with them were scheduled. Data were collected through a once off semi-structured interview with the first author (DR) who was also the primary investigator for the project. Interviews were conducted with five facility managers and five MHCUs at the participants’ place of work (in case of facility heads/managers) or dedicated health care facility (in case of service users). Interviews were approximately 60 min in duration and audio recorded. Participants chose their own pseudonyms to anonymise their identity and ensure confidentiality.

**Table 1.** Descriptive information of the sub-study population.

Facility Managers	Occupation	Period serving in public health care	Period serving at current facility
Mrs X	Operational manager	33 years	16 years
Lollypop	Professional nurse	21 years	4 years
Nana	Professional nurse	4 years	2 years
Avy	Operational manager	34 years	5 years
Beyonce	Professional nurse	22 years	12 years
Mental health care service users	Age	First language	Employment status
Charmy	58 years	Afrikaans	Unemployed
Heksie	43 years	Afrikaans	Nail technician
Gavi	51 years	Afrikaans	Unemployed
André	41 years	Afrikaans	Unemployed
Khosinathi	33 years	isiXhosa	Unemployed

### Data analysis

Audio recordings were transcribed and loaded onto Atlas.ti 22. We used Thematic Analysis (Braun & Clarke, 2006) to analyse the information obtained from the participants.

## Findings

From the interviews with participants and subsequent data analysis, a number of themes emerged that addressed the aim of the project. The themes with supporting narratives are presented in Table 2:

**Table 2.** Themes that emerged during analysis of participants’ experiences.

Themes
The integration of MH care into routine primary health services poses challenges to treatment delivery.
The integration of MH care into routine primary health services poses challenges to patients who come for treatment.
Resegregating care as a solution?

*Note*. MH = Mental Health.

### The integration of mental health care into routine primary health services pose challenges to treatment delivery

Many of our participants indicated that the integration of MH care into routine care at primary health level creates challenges for treatment delivery.

Mrs X explained how service delivery has changed over the past 16 years since she started working at the PHC facility. She highlighted that there used to be a dedicated MH care sister in the past, who was responsible for primarily attending to MH service users. Since the integration of care services, however, the sister had resigned and dedicated care delivery subsequently collapsed:

Interviewer: *But you are talking about the past 16 years since you got here and how things were when you got here and that kind of leads into my next question of – has there been any evolution? Have things changed since 16 years ago?* (translated from Afrikaans to English)

Mrs X: *Yes, I’m saying that there was a section that was run by the psychiatric sister and she JUST looked after those psychiatric people and we held the fort here with the other chronic diseases, with mother and child and so on, but then we were told that we have to integrate and the sister has now gone on retirement and what, what, what, then everything just fell flat.* (Mrs X, facility manager; translated)

Beyonce explained how the integration process has resulted in many illnesses currently being treated within the same consulting room. This negatively impacts valuable time required to engage and treat MH care service users. She also noted how she was the dedicated service provider to MH care users in the past. However, since integration, patients are seen when it is their appointment, and this for a short duration of time:

Interviewer: *And how much time do you have for your psychiatry patients?* (translated)

Beyonce: *Little, too little. Because look, now they have now integrated it into the chronic room, with the ideal clinic aspect. So in that chronic room it’s psychiatry, it’s TB, it’s HIV, it’s epileptic, all those chronic conditions in one. This means you go according to an appointment schedule. So, you now have to go according to that appointment, and each appointment is about 10 or 15 minutes, then that patient has to be out. In the past when it didn’t exist, I could make time for them [referring to psychiatric patients] and I could talk to them. Everyone in the clinic knew the psychiatry patients belonged to me. If they, if one of them comes, then they come and call me. Sister, that person is here. Then I know that person, I gave them special treatment. That person’s file is now being drawn and I’m going to see you now and I’m going to make time for you now. Now this is not the case – the patient must be seen when his appointment is there and I can only spend that amount of time with you. (Beyonce, facility manager; translated)*

Mrs X shared a similar opinion to that of Beyonce. Mrs X feels that MH service users have been neglected since the integration of primary care:

Interviewer: *And how do you feel about this integrated care, which is now throwing chronic, medical, geriatrics and mental health together? (translated)*

Mrs X: *What can I say, its just a, a what can I say, we, we, we leave some people out. The most important people who are left out here are the mental health, the geriatrics and the eye patients. Those patients are really neglected, those three groups. (Mrs X, facility manager; translated)*

Lollypop reported that their facility is constantly busy. She has to quickly attend to service users to make sure all health care users are seen by the end of a day. The rushed service delivery, however, led her to question the quality of care she is providing:

*I feel, I feel. Yoh, we do incomplete work. That’s how I feel like. And then at the end of the day, you look in your register and feel like yoh, I’ve seen so many clients today but what I’m asking myself is, what quality of care did I give today? Because people will look in my register and they will say, “Haibo, you only saw so and so and so” and I’ll say yes. . . forever we are in a rush. We want to get done with the work. The waiting area is full. We want to get the people out of the facility. (Lollypop, facility manager)*

Mrs X concluded her experiences of the integration of MH care into routine PHC. It is her opinion that integrated health care is not suitable for effective MH service delivery:

*Now as I say, only the integration . . . integrated e, e, e that thing we are doing now is working but just not for the psychiatric people. I’m sorry to say this, but for me it doesn’t work. The psychiatric service . . . the psychiatric service that is now integrated.* (Mrs X, facility manager; translated)

### The integration of mental health care into routine primary health services poses challenges to patients who come for treatment

This theme aims to illustrate that the integration of MH care into routine care at primary health level creates challenges for MHCUs who come for their treatments.

Khosinathi explained how he would arrive early in the mornings for his treatment. However, he experienced long waiting periods that compelled him to leave before receiving his treatment. Khosinathi also alluded to feeling that health care authorities neglected to consider ‘them’ in this process:

*Oh no, sometimes it’s just, it affects me so much after some time that I just want to walk away from the clinic. Then it feels like I’m sitting for too long. And sometimes it’s too long, then it’s already one hour, you sit and wait here like this. I came early in the morning already. That, we don’t think . . . they sure didn’t think. That’s probably so.* (Khosinathi, MHCU; translated)

Gavi is motivated to attend appointments and adhere to treatment, but the waiting time is stressful for him and it exacerbates his illness. He explained:

Interviewer: *What happens if you have to stand here and wait? (translated)*

Gavi: *That’s something funny, sir . . .* (translated)

Interviewer: *Yes?* (translated)

Gavi: *It works on me, sir, because it’s my date, the waiting doesn’t make right with me, sir. Everywhere I go I have to wait. There, when I almost stand behind, I get sicker and then I have to take pills now I have to take pills at home, then I get those eye problems again. (Gavi, MHCU; translated)*

Heksie expressed irritation and frustration at the long queues and many service users around her on treatment days:

*And I, you know, it sounds like I’m looking for special treatment or something, but half the people sitting here come for shit. I have been seeing this for the past five years. There are 110 women here who are pregnant – some of them are little girls of 12 years old. It’s rows and rows of people. You never know where you stand when you walk in here for the day. You never know what time you’re going to walk out here.* (Heksie, MHCU; translated)

Charmy told of her experiences when attending her care facility. She has to wait outside the facility for long periods until health care workers can attended to her. Furthermore, it is her opinion that service delivery is slow at the under-staffed facility.

Interviewer: *So then you have to stand outside? (translated)*

Charmy: *You have to stand outside and wait before you can come in here now. The staff is also too few here. I feel that the staff is too few, because you wait too long for them to finish with one patient in there. And they’re a little slow – they’re a little slow. . .. You can’t sit out here for an hour, two hours and wait for them to finish now.* (Charmy, MHCU)

Heksie also experienced having to wait in a queue when collecting medication. Despite being a known MHCU at the facility she needed to re-explain her reason for her visit to attending staff on a monthly basis. She feels that she is not a priority at the facility. Furthermore, she provided subtle hints of exposure to stigmatisation that is related to mental illness:

Heksie: *I understand we have to fall in the same line every month to get our pills but then to go through all the questions about why are you here? Then you have to tell her, no I’m here to get my pills, and then she asks, what’s the pills? And then you have to say, my pills for bipolar, in front of everyone. So I say every month I tell everyone there I’m bipolar – everyone who can hear. And I don’t care – people can know. Because I don’t see it as a, as a sin or whatever, it’s not – I didn’t choose it. It chose me. And I have learned to live with it, but if it can be made easier to just be able to get my stuff every month that I can cope with, that would be much better. It will really, it will help just so much, help so much more . . .*

Interviewer: *But that the experience for you here is that sometimes it goes slowly or stops*. (translated)

Heksie: *Yes. It feels as if one is not a priority.*

Interviewer: *Yes. Someone who is bipolar is not a priority.* (translated)

Heksie: *You are crazy.* (Heksie, MHCU; translated)

### Resegregating care as a solution?

This theme illustrates participants’ views that the segregation of MH care from routine care at primary health level may facilitate access and provision for MH care for service users and facilities. It draws on narratives of facility managers as well as MH care service users.

Mrs X explained that MH care services were facilitated in the past when care was dedicated to a specific nurse. She added that care had diminished since the dedicated nurse no longer provides MH care:

*Because I have been at the facility for 16 years now and when I came here, it was – but at that time it was not yet integrated. Then there was a, a, a dedicated nurse who did it and it went very well. And those dedicated nurses also went to the different villages. She also had her special people there who she went to visit. Like now everything has just disintegrated with the, collapsed because of the integration.* (Mrs X, facility manager; translated)

Lollypop reported that, as facility manager, she strategically removed MHCUs from the integrated care system – as this method was effective. Lollypop explained that MHCUs now have separate appointments in the afternoons when the facility is less busy and that reduces waiting time for service users:

*So what we’ve done is, we, this was like two years ago. I think 2020, yes. They used to full into our mainstream. Mainstream meaning our chronics. Irrespective what treatment you’re coming for. And I saw that time that, this isn’t right because if I am, for example, a high blood pressure client, I’m coming for my treatment. I have an appointment. You doing exactly the same that you doing for me, you doing for the mental health care client. Meaning, the way we make appointments, the way we’ve seen our clients, we generalise basically. So, I had a, I separate them in terms of appointments. So we gave them afternoon slots. We asked them before the time, listen here, afternoon slots, can we accommodate you? Can we book you? Because that time we know the facility is more or less quiet. The morning session, mostly the appointments is done in the morning. So if you come in the afternoon, a specific time we give you, then it will be no waiting time for you.* (Lollypop, facility manager)

Gavi told the interviewer that he feels helped when he gets a set appointment in the afternoons, with reduced waiting periods to receive his treatment:

Interviewer: *And when you have come here recently, do they help you quickly?* (translated)

Gavi: *Yes, sir, they help me, they no longer make me wait for other people who come here. I get a date that I come here, then I don’t wait so long for other people. I said once [I] come in the afternoons then there are not so many people.* (Gavi, MHCU; translated)

Heksie received her treatment at a facility where the MH care treatment has not been strategically segregated and remains part of integrated health provision. She, however, suggested that her treatment facility introduce appointment-based treatment for MHCUs. She feels that MH care would be facilitated in this manner:

*It just has to be easier to, even if they then try to say okay, listen, say once a month or on a Wednesday, people with mental health can come and collect their pills between so and so late. They’re going to come and get files, pills, stuff like that. It will already be much easier.* (Heksie, MHCU)

## Discussion and conclusion

To our knowledge, the presented research is the first of its kind in the public primary health sector in the DBNLM district. The study provides first insights into facility managers’ and service users’ views of integrated MH care at PHC level in the rural parts of the Eastern Cape of South Africa. While MH care services have been expanded and integrated into PHC over recent years, the system may not have been as streamlined as reported in other parts of the country. Can health care facilities offer what service users may need during their visits to this ‘one-stop shop’? Can optimal levels of MH, wellness, care, and rehabilitation be reached through integration of care in this part of the country?

Integrated healthcare may be helpful – especially during initial stages of assessment and treatment of patients or for screening for comorbidities. The findings from our study however suggest that in the absence of adequate resources; integration of MH into PHC can pose various challenges to facilities, health care providers, and MHCUs. Integration of care, it appears, may have contributed to the phasing-out of the, already scarce (De Kock & Pillay, 2016), dedicated MH nurses who previously cared for MH patients. MH service users now have to wait in the same queue as others with acute or chronic health conditions before they can be attended to by the next available health care worker. As a result, facilities are burdened with high service user load, creating long queues and waiting times before treatment. Health care workers frequently have to rush to get through their patient load, which makes them question the quality of care provided. Some health care providers go so far as stating that their rushed work leads to neglect and ineffective treatment.

One of the key arguments in favour of integrating MH into PHC in South Africa, as we have shown, is that having patients with MH problems as part of the general pool of patients may reduce stigma. This is essentially an argument about the potential benefits of intergroup contact. Literature on intergroup contact, however, shows that for social integration and acceptance to succeed, as when people are grouped together across various domains of society, certain core conditions must be met, for example, status equality across groups and support for contact from those in authority are key (Dixon et al., 2010; Pettigrew, 1998). In the case of this health authority, and, likely, many others, these facilitating interventions are not in place. It seems evident that the integration of care at rural level may have overstretched the capacity of care that facilities and health workers can offer. Service users experience feeling othered and labelled as a result of their mental illness. Furthermore, MH service users experience the queues as distressing; they feel non-prioritised and annoyed at having to wait long periods before being attended to. Some service users experience these long waiting times as increasing their levels of distressing symptoms. Our study supports the work of other local literature by Swartz and MacGregor (2002) in showing that certain populations, such as MH patients, may not necessarily benefit from integrated health care modalities as others with other chronic conditions may, given current resource constraints. The experiences reported in our study show that providing open, unstructured, first-come-first-serve health care at PHC level may in fact hinder and diminish MH care access to the vulnerable populations in low-resourced contexts.

We are aware that our findings contrast with much of current wisdom on service planning. Literature suggests that silo or independent treatment modalities can be problematic to overall service delivery and management of patients (Mokitimi et al., 2019). Yet facility managers in these constrained circumstances have observed that segregating MH care from physical treatment, as in the past, may deem more effective for health care provision and reception. This does not imply removing multi-disciplinary referral pathways or intervention, but rather shows a practice to some degree of separating MH care from physical health care. As a result, some facilities strategically structured their service provision in a manner which looked like resegregation. Resegregating mental and physical health into two different lines of care may portray that one is more important than the other. It may also mimic silo treatment modalities that may seem to neglect holistic health care. However, the change towards resegregated treatment delivery contributed to service users receiving their MH treatment during fixed appointments when the clinics are more quiet, which allowed health workers to longer attend to patients’ presenting needs. For patients, shorter queues and waiting times facilitated their experiences of care reception and wellness. Perhaps a possible way forward is that MH service users can have different appointment slots for their physical and mental treatment when needed/desired. In such case patients may experience that their health needs are attended to at set times and limit the distress caused by having to wait in uncertainty for prolonged periods of time before receiving care.

Our study suggests that the integration of MH care with physical care at PHC level in this part of the country may not be as effective as may be assumed. It is well established in the field of disability inclusion more broadly, that simply having people in the same place is not the same as inclusion. For example, placing children with disabilities in a classroom with others, but without appropriate support where necessary, may lead to increased exclusion and marginalisation than providing specialised services (Bantjes et al., 2015). Disability inclusion in health, as the recent World Health Organization (WHO) Global Report on Health Equity for Persons with Disabilities (World Health Organization [WHO], 2022) emphasises (and this includes inclusion of people with what the WHO would term ‘psychosocial disabilities’ or mental disorders), requires broader institutional and attitudinal change. It is not just a question of having people in the same place or the same queue. The question arises if segregated care may be more effective; or if better integrated care should be facilitated. Inclusion requires resourcing, support and attitudinal change, as well as sensitivity and responsiveness to specific care needs. In a resource constrained environment like the one we have studied, thoughtful integration may well be an ideal, *de facto* segregation of a sort may be more practical as an approach. Facilities and health care providers must be supported to be cognisant of their specific structures, resources, and service provider/user needs. Generalised integration of MH treatment with physical care should be approached with caution unless there is wider scale provisioning and greater organisational change. Adjusting to changes may be difficult to accommodate for facilities, health care providers, and service users. Facilities, health care workers, and patients in our study showed a preference, in their context, for a degree of segregation and specialisation of services. Some of the preference to status quo at some time may have been related to adverseness to change; but the current operational method has been in place for many years. Marais and Petersen (2015) have similarly highlighted the importance of health system governance changes as fundamental to integration efforts; where such changes are not in place, integration may, paradoxically perhaps, do some harm. It is important to stress that we are not suggesting that integrated care is not desirable or helpful; what we have found however is that with limited infrastructure and resources to support integrated care, many practical challenges may arise. Full and successful integration of MH care integration into PHC requires more than a simple integration of queues, but requires the comprehensive assessment of the circumstance in which health care is delivered as a whole.
